# Digital Health Intervention (SANGYAN Podcast) to Enhance Knowledge Related to COVID-19 and Other Health Conditions: Protocol for an Implementation and Evaluation Study

**DOI:** 10.2196/41175

**Published:** 2025-01-20

**Authors:** Ashish Joshi, Surapaneni Krishna Mohan, Apurva Kumar Pandya, Ashoo Grover, Sofia Rani Saggu, Saravanavel Kalpana Revathi, Shruti Sharma

**Affiliations:** 1 School of Public Health University of Memphis Memphis, TN United States; 2 Panimalar Medical College Hospital & Research Institute Chennai India; 3 Parul Institute of Public Health Vadodara India; 4 Indian Council of Medical Research New Delhi India; 5 Foundation of Healthcare Technologies Society New Delhi India; 6 SMAART Population Health Informatics Intervention Center Foundation of Healthcare Technologies Society - Panimalar Medical College Hospital & Research Institute Chennai India

**Keywords:** podcast, human-centered behavior, pandemic, coronavirus, intervention, digital health, usefulness, effectiveness, usability

## Abstract

**Background:**

Podcasts are an unconventional method of disseminating information through audio to the masses. They are an emerging portable technology and a valuable resource that provides unlimited access for promoting health among participants. Podcasts related to health care have been used as a source of medical education, but there is a dearth of studies on the use of podcasts as a source of health information. This study will provide new perspectives by implementing the SANGYAN podcast, which contains information about COVID-19 and other health conditions.

**Objective:**

The study aims to determine the usefulness and effectiveness of the SANGYAN podcast as a digital health intervention to address misinformation related to COVID-19 and other health conditions among individuals in Chennai, Tamil Nadu, India.

**Methods:**

An implementation and evaluation study will be conducted with 500 participants from the Panimalar Medical College Hospital & Research Institute (PMCHRI) and Rural Health Training Centre in Chennai. Among individuals aged 18 years and older, those residing in the selected urban and rural settings who visit the outpatient department of the PMCHRI and Rural Health Training Centre will be recruited. For participants who consent to the study, their sociodemographic details will be noted and their health literacy will be assessed using the Rapid Estimate of Adult Literacy in Medicine scale. Once the participants have listened to the podcast, the usability, acceptance, and user satisfaction of the podcast will be assessed. Descriptive analysis will be used for continuous variables, and frequency analysis will be used for categorical variables. Bivariate analysis will be conducted to understand the correlation of sociodemographic features in response to perception, usefulness, acceptance, and user satisfaction of the podcast. All analysis will be performed using SPSS (version 24), and the results will be reported with 95% CIs and *P*<.05.

**Results:**

As of December 2024, the SANGYAN podcast has been launched for voluntary usage in the PMCHRI.

**Conclusions:**

The finding from this research project will aid in the development and implementation of data-driven, evidence-based, and human-centered behavior change interventions using podcasts to address public health challenges among populations living in diverse settings. This would also help in enhancing the acceptability of podcasts as a source of health-related information.

**International Registered Report Identifier (IRRID):**

DERR1-10.2196/41175

## Introduction

### Background

The term “podcast,” coined in 2004, is derived from the terms iPod and broadcast. It was chosen as the word of the year by the New Oxford American Dictionary in 2005 [1,2]. It is an unconventional means of distributing information to the general public via audio. Podcasting is a relatively new portable technology. It has established itself as a reliable channel for knowledge and information exchange. Podcasts are classified under Web 2.0 tools among other tools such as wikis and blogs, which comprise the latest generation of web-based collaboration [3]. Technology-enabled approaches to managing lifestyle have begun to emerge with the increased development and use of electronic media. Public health practitioners are now using mobile health technology to deliver health-related interventions [4]. However, there are hurdles to offering web-based interventions since they often need research participants to have a mobile phone, a certain level of education, and the capability of properly comprehending textual material [5]. The development of audio or video material for an audience that wants to listen to what they want, when they want, where they want, and how they want is the heart of podcasting [6].

India has reported a significant shift in the number of podcast users over the years. A report states that India holds the third-largest podcast user population after China and the United States, with over 57.6 million users [7]. Podcasting is becoming increasingly popular due to its ability to communicate health-related data and information for educational reasons [3]. Podcasting represents a largely untapped conduit for promoting health information to the general public with internet access and those who are not comfortable seeking face-to-face knowledge, treatment, and guidance. The COVID-19 pandemic played a significant role in increasing the consumption of podcasts by 29.3% in India [8]. Podcast use in entertainment, lifestyle, health, society, and culture is widespread according to a Spotify report. While in regard to health care [9-11], podcasts in India have seen increasing use in medical education and training, but not in other aspects of health care [12]. There is a dearth of research showing the use of podcasts to address misinformation, especially in an Indian context. A medium like a podcast provides screen-free alternatives even during the COVID-19 pandemic’s restrictive environment. Podcasts are becoming increasingly popular among the younger population as a source of entertainment, self-improvement, and awareness.

### Why Use a “Podcast” to Deliver Public Health Intervention?

According to user control theory, allowing flexibility boosts learning as compared to traditional instruction such as print. Podcasting may be more effective than the web because such a platform can decrease cognitive load [13]. Podcasts can encourage users’ positive knowledge, attitude, and practice by allowing them to listen to the podcast anywhere, anytime. Podcasts promote knowledge dissemination by allowing podcast channel owners to communicate knowledge even when movement is restricted (eg, during a pandemic). The most prominent reason for the podcast boom is the connection between the host and listener. Podcast technology is a valuable resource that provides unlimited access for promoting health among participants. It also plays a significant role in public health and global health, where challenging and critical situations demand simple, effective, and concise information delivery. Numerous studies have examined the acceptability or feasibility of this modality for learning; however, limited studies have focused on podcast use for knowledge sharing [14]. Also, journals such as The *New England Journal of Medicine* [13] and *Lancet* [15] currently use podcasts to support medical and nonmedical information dissemination.

### Need for the Study

The COVID-19 pandemic has shown that misinformation spread via social media and other digital platforms is a more significant threat to global public health than the virus itself [16]. Studies show that female individuals are more likely to accept misinformation than male individuals. A higher education level also decreases the possibility of accepting misinformation. Thus, older people with a higher level of education are less likely to accept misinformation [17,18].

This research will provide new perspectives through implementing the SANGYAN podcast, which contains information about the COVID-19 pandemic and other health conditions. The research project’s findings will directly benefit individuals and researchers in developing tailored intervention models aimed at addressing misinformation.

### Study Objectives

The study aims to determine the usefulness and effectiveness of the SANGYAN podcast as a digital health intervention to address misinformation related to COVID-19 and other health conditions among individuals in Chennai, Tamil Nadu, India.

## Methods

### Study Design and Population

An implementation and evaluation study will be conducted at the outpatient department of the Panimalar Medical College Hospital & Research Institute (PMCHRI) and Rural Health Training Center (RHTC) in Chennai. The participants will be recruited through convenience sampling in a paper-based format, by the researchers or trained data collection team from the RHTC and PMCHRI. A total of 500 individuals (250 each from the PMCHRI and RHTC) will be enrolled for the study. The data will be collected at a single time point by administering the study questionnaire to the eligible study participants. For participants who consent to the study, their sociodemographic details will be noted and their health literacy will be assessed using the Rapid Estimate of Adult Literacy in Medicine (REALM) scale. Once the participants have listened to the podcast, the usability, acceptance, and user satisfaction will be assessed.

The eligible study participants will comprise individuals (1) aged 18 years and older, (2) residing in the selected urban and rural settings who visit the outpatient department of the PMCHRI and RHTC, and (3) consenting to participate in the study. Individuals with any mental or physical challenges that might affect their ability to participate in the study will be excluded.

### Variable Assessment

#### Sociodemographic Profile

Sociodemographic data will be gathered, including participants’ age, gender, income level, education level, employment status, occupation, region of residence, marital status, parenthood, and religion.

#### Health Literacy

The REALM scale is one of the most widely used tools to measure health literacy. Statistically, the REALM scale appears to provide a highly reliable data [19]. The study will use this scale as a screening instrument to assess an adult patient’s ability to read common medical words. It is designed to assist medical professionals in identifying patients with poor literacy skills [20].

#### Use of Podcast or Other Mediums for Health Information

This measure will help us gather data on prior use of podcasts or other sources by participants to gather health data.

#### Usability

A Likert-scale System Usability Scale (SUS) survey will assess user acceptance. The SUS is a 10-item questionnaire with 5 response options, ranging from strong agreement to strong disagreement on a scale of 0 to 4, for each question. The total score will be calculated by adding the converted responses for each user and multiplying that total by 2.5. This will restore the range of possible values to 0-100 instead of 0-40 [21].

#### Client Satisfaction Questionnaire-8

The Client Satisfaction Questionnaire-8 (CSQ-8) is an 8-item measure of client satisfaction with services. The items for the CSQ-8 were selected on the following basis: ratings on information seeking by participants for a number of items that could be related to client satisfaction and a subsequent factor analysis. The CSQ-8 is unidimensional, yielding a homogenous estimate of general satisfaction with services [22].

### SANGYAN Podcast

The SANGYAN podcast comprises audio content that will be delivered over a network via a free subscription.

The steps for developing the SANGYAN podcast are as follows: (1) file production, (2) podcast publication, (3) podcast delivery, and (4) podcast playback. File production involves planning, writing, recording and editing content, and file compression. Recording requires hardware like a recording microphone, and editing requires software like Audacity (The Audacity Team) and Premiere Adobe Pro (Adobe). The feed would be a simple XML file that lists the location of the podcast’s COVID-19 episodes. The meta-tagging will have file information like the producer details, date of publication, title, and description of each episode. The RSS feed then will be posted to the web server of SMAART Rapid Tracker [23]. The podcast is hosted by the Foundation of Healthcare Technologies Society with a team of researchers that curate evidence-based content. Listeners will be able to subscribe, access, and download the podcast file.

### Data Collection, Data Entry, and Quality Assurance

Data will be collected using a structured questionnaire. The questionnaire will be presented to the participants in the local Indian dialects to help increase the usefulness and generalizability of the study data. Data collection and data entry will be performed by a team of data collectors and data management personnel. To ensure efficiency and high-quality data collection and processing, the following data management protocol is in place: a clearly defined study manual, a well-trained team of data collectors, weekly meetings with the research team, weekly data checks, maintenance of study participants contacts, and data instrument logs.

### Expected Outcomes

The proposed research study will help explore the usability and acceptability of a health-related podcast. Further, it will help evaluate the SANGYAN podcast’s usability as well as satisfaction toward information delivery through a podcast. The results of the study will help design and develop a podcast platform that can deliver health educational information to facilitate podcast acceptance in varied settings. The expected study outcomes include the acceptability of using a digital health podcast to obtain tailored and evidence-based health information related to COVID-19 and other health conditions.

### Data Analysis Plan

The gathered data will be presented in tables comprising the recorded characteristics of all variables. These tables will serve the purpose of data quality control to find inconsistencies in the data patterns and outliers or any missing data. Descriptive analysis will be conducted to report the means and SDs of the continuous variables (such as SUS scores), and frequency analysis will be conducted for the categorical variables (such as CSQ-8 scores and data on the use of podcasts and other mediums for health information). Bivariate analysis will be conducted to understand the correlation of sociodemographic features in response to perception, usefulness, acceptance, and user satisfaction of the podcast. All analysis will be performed using SPSS (version 24; IBM Corp), and the results will be reported with 95% CIs and *P*<.05*.*

### Project Timeline and Milestones

A detailed study timeline is presented in Table 1.

**Table 1:** Project timeline and milestones.

Task	Month														
	1	2	3	4	5	6	7	8	9	10	11	12	13	14	15
Review of the literature, initial designing, and planning of the study	✓														
Development of study proposal and ethical approval	✓														
Approval of the study proposal	✓														
Development of survey items and the questionnaire	✓														
Review and revision of the questionnaire by the research team		✓													
Recruitment and training of the data collector team		✓													
Recruitment of the target sample		✓	✓	✓	✓	✓	✓	✓	✓	✓	✓	✓	✓		
Data analysis														✓	
Results write-up and preparation of the manuscript														✓	
Dissemination														✓	✓

### Ethical Considerations

This study (protocol PMCHRI-IHEC-056) gained approval from the PMCHRI Institutional Human Ethics Committee (Central Drugs Standard Control Organization Registration ECR/1399/Inst/TN/2020) in February 2022, with approval PMCH&RI/IHEC/2021/078 (dated February 18, 2022).

The institutional review board–approved informed consent form will be administered by the research team to the eligible individuals for the study. The research team will explain the study, the time commitment necessary, and the advantages of the study results to participants. Those willing to participate and give their consent will be enrolled in the study. Written informed consent will be obtained in both English and local Indian dialects. The data gathered will be stored securely, ensuring data privacy and confidentiality. No compensation will be provided.

### Dissemination

The study’s findings will be disseminated through peer-reviewed publications and national and international conference presentations. Results will also be disseminated to the local community health leaders, state officials, and policy makers for data-driven, evidence-based, and informed decision-making.

## Results

As of December 2024, the SANGYAN podcast has been launched for voluntary usage in the PMCHRI.

## Discussion

Podcasts are rapidly being used as a tool for information distribution by a wide range of organizations and associations, including medical and dentistry schools, research institutes, and scientific journals. There is a need for robust strategies to boost podcast acceptability and provide community-specific information on misconceptions about public health concerns. The study would help assess the acceptability of podcasts as a source to obtain tailored and evidence-based health information.

The study findings will elucidate the association of the acceptance and usability of podcasts with various sociodemographic factors such as education level, gender, and occupation, for which there is a dearth of data in the Indian context. The findings will also provide insight into people’s awareness of podcasts and other digital sources of health information prior to the study.

The rollout of the protocol may see some limitations, in that it is a one-time implementation of the SANGYAN podcast and adherence is not assessed. Another limitation could be in its implementation within a limited geographical setting; thus, future studies regarding the usability, acceptance, and adherence can be carried out at a larger scale across various states in India.

Studying the spread of misinformation (or infodemics) in India remains critical considering the booming use of social media in the wake of the COVID-19 pandemic. India, along with 132 member states of the United Nations, has endorsed fighting infodemics especially during COVID-19 [24]. This study relevant to this endorsement, as the study findings will contribute to the development and implementation of data-driven, evidence-based, and human-centered behavior change interventions using podcasts to address public health challenges among populations living in diverse settings. Further, this study would aim to expand the use of the SANGYAN podcast as source of evidence-based health information for the public.

## Abbreviations

CSQ-8: Client Satisfaction Questionnaire-8
PMCHRI: Panimalar Medical College Hospital & Research Institute
REALM: Rapid Estimate of Adult Literacy in Medicine
RHTC: Rural Health Training Centre
SUS: System Usability Scale

## Data Availability

The data supporting this study’s findings are available on request from the corresponding author.
